# Evolutive analysis of the SOFA score in critically ill massive burn patients during their stay in the ICU

**DOI:** 10.1186/cc14714

**Published:** 2015-09-28

**Authors:** Edvaldo V de Campos, Luciano Cesar P Azevedo, Marcelo Park

**Affiliations:** 1 Hospital das Clínicas, University of São Paulo Medical School, São Paulo, SP, Brazil

## Introduction

It is estimated that multiple organ dysfunction could be responsible for 80 % of mortality in critically ill burn patients, mainly related to sepsis. However, there are no recommendations for monitoring organ dysfunction during the stay of burn patients in the ICU.

## Objective

Our aim was to analyze and characterize organ dysfunction by the Sequential Organ Failure Assessment (SOFA) score in massive burn patients during their stay in the ICU with the hypothesis that survivors diverge from nonsurvivors during the length of stay in the ICU.

## Methods

Retrospective cohort study employing data collected from May 2005 to April 2010 at an ICU specializing in burn patients at a teaching hospital. All patients admitted during this period were included. Data for physiological and epidemiological variables were collected at admission. During the ICU evaluation, the total SOFA score with its components were recorded from the first day to the seventh day. It was also recorded on the 14th, 21st and 28th days if the patient stayed in the ICU. The clinical outcomes collected were the ICU length of stay, hospital length of stay, and ICU and hospital mortality.

## Results

One hundred and sixty-three consecutive patients were studied (male: 71 %), with median age of 34 (25, 47) years and a hospital stay of 29 (11, 50) days. Incidence of inhalation injury was 45 % and total burn surface area (%) was 29 (18, 43). The total SOFA score at admission in survivor patients was 1 (1, 4) and in nonsurvivors was 7 (4, 9) (*P *<0.05). Significant difference related to the total SOFA score was found during all other days analyzed. The analysis in the first 7 days with the receiver operating characteristic curve of worst individual organ dysfunction quantified through the total SOFA score and partitioned SOFA score showed good capacity to discriminate survivors and nonsurvivors between the respiratory, cardiovascular, renal and hematological components. Hepatic and neurological components did not present a good performance.

## Conclusion

In our study, organ dysfunction quantified by total SOFA score and respiratory, cardiovascular, renal and hematological partitions was different between survivors and nonsurvivors during the ICU evolution.

